# Galectin-3 Functions as an Alarmin: Pathogenic Role for Sepsis Development in Murine Respiratory Tularemia

**DOI:** 10.1371/journal.pone.0059616

**Published:** 2013-03-20

**Authors:** Bibhuti B. Mishra, Qun Li, Anthony L. Steichen, Brandilyn J. Binstock, Dennis W. Metzger, Judy M. Teale, Jyotika Sharma

**Affiliations:** 1 Department of Microbiology and Immunology, University of North Dakota School of Medicine and Health Sciences, Grand Forks, North Dakota, United States of America; 2 South Texas Center for Emerging Diseases and Department of Biology, University of Texas at San Antonio, San Antonio, Texas, United States of America; 3 Albany Medical College, Albany, New York, United States of America; University of São Paulo, Brazil

## Abstract

Sepsis is a complex immune disorder with a mortality rate of 20–50% and currently has no therapeutic interventions. It is thus critical to identify and characterize molecules/factors responsible for its development. We have recently shown that pulmonary infection with *Francisella* results in sepsis development. As extensive cell death is a prominent feature of sepsis, we hypothesized that host endogenous molecules called alarmins released from dead or dying host cells cause a hyperinflammatory response culminating in sepsis development. In the current study we investigated the role of galectin-3, a mammalian β-galactoside binding lectin, as an alarmin in sepsis development during *F. novicida* infection. We observed an upregulated expression and extracellular release of galectin-3 in the lungs of mice undergoing lethal pulmonary infection with virulent strain of *F. novicida* but not in those infected with a non-lethal, attenuated strain of the bacteria. In comparison with their wild-type C57Bl/6 counterparts, *F. novicida* infected galectin-3 deficient (galectin-3^−/−^) mice demonstrated significantly reduced leukocyte infiltration, particularly neutrophils in their lungs. They also exhibited a marked decrease in inflammatory cytokines, vascular injury markers, and neutrophil-associated inflammatory mediators. Concomitantly, in-vitro pre-treatment of primary neutrophils and macrophages with recombinant galectin-3 augmented *F. novicida*-induced activation of these cells. Correlating with the reduced inflammatory response, *F. novicida* infected galectin-3^−/−^ mice exhibited improved lung architecture with reduced cell death and improved survival over wild-type mice, despite similar bacterial burden. Collectively, these findings suggest that galectin-3 functions as an alarmin by augmenting the inflammatory response in sepsis development during pulmonary *F. novicida* infection.

## Introduction

Sepsis results in 750,000 hospitalizations every year in the US and is the second leading cause of mortality in patients admitted to intensive care units [1]. Pulmonary infections, in turn, are a major cause of sepsis [2]. However, the mechanisms responsible are not well understood. This is underscored by a lack of effective therapeutics against this immune disorder despite more than two decades of active research. Our recent studies have shown that pulmonary infection of mice with fully virulent *Francisella tularensis* as well as the murine model organism *F. novicida* (F.n.) a Gram negative bacterial pathogen, leads to development of severe sepsis characterized by hyperinflammation, T cell depletion, and extensive cell death in systemic organs [3]–[5]. We are thus using a murine inhalation model of F.n. infection to understand the mechanism/s responsible for pulmonary infection-induced sepsis development. Intriguingly, this pathogen is not known to produce any exotoxin, which can account for the lethality of this infection. Moreover, the lipid A of Francisella LPS does not stimulate TLR4 and is thus hypo-inflammatory [6]. Studies from our and other laboratories have shown that extensive tissue damage and wide-spread cell death is a hallmark of Francisella infection, regardless of the bacterial strain [4], [7]–[10]. Additionally, our studies show that Francisella infected macrophages are defective in clearance of dead cell debris, a process termed efferocytosis, leading to accumulation of these dead cells and their contents [11]. We thus hypothesized that in the absence of any bacterial toxins, host endogenous molecules released from these dead or dying cells contribute to the inflammatory response culminating in sepsis development during respiratory infection with Francisella.

Alarmins are host endogenous factors which perform homeostatic functions when contained within cellular compartments [12]. However, under pathological conditions, these molecules can be released either passively from dead cells or actively via non-classical secretion pathways [13]. Once in the extracellular milieu, they exhibit immune modulatory properties such as induction of pro-inflammatory cytokines, immune cell chemotaxis, and regulation of cell death [12]–[14]. The overt inflammation during sepsis is primarily a result of the interaction between innate immune receptors with pathogen derived molecules (Pathogen Associated molecular patterns (PAMPs) and alarmins. PAMPs and alarmins together constitute Danger-Associated Molecular Patterns (DAMPs). The interaction of Toll-Like receptors (TLRs) as well as NOD-Like receptors (NLRs) with pathogen derived PAMPs during sepsis has been studied extensively (reviewed in [15], [16]. However, the recognition of self-molecules (alarmins) by signaling receptors and the concomitant inflammatory response is an area of research which is still in its infancy. Moreover, in a complex immune disorder like sepsis which is an interplay of several host immune pathways such as the coagulation system, complement cascade and even the autonomic nervous system [15], it is likely that several alarmins are involved at the intersections of these pathways. Thus, identification of novel alarmins may aide in understanding this complex disorder and may present additional targets for effective therapeutics. As sepsis developed during pulmonary infection with Francisella is associated with extensive cell death in lungs and other systemic organs, we sought to identify novel alarmins that might be released during this infection and may contribute to disease development.

Galectins constitute a soluble mammalian β-galactoside binding lectin family which play homeostatic roles in regulation of cell cycle and apoptosis, as well as display inflammatory and immune modulatory activities in various pathological conditions [17]–[20]. Previous studies have implicated galectin-3 in regulation of various inflammatory conditions including endotoxemia and airway inflammation [21]–[23]. In this study we show that galectin-3, a mammalian galactoside binding soluble lectin is upregulated and released in lungs of mice undergoing lethal respiratory infection with F.n. but not in mice vaccinated with an attenuated mutant strain of the bacteria that protects these mice from an otherwise lethal challenge. We thus hypothesized that galectin-3 exacerbates the inflammatory response during lethal infection. The outcome of this study, with use of galectin-3 deficient mice, shows that galectin-3 plays the role of an alarmin in Francisella infection induced sepsis development.

## Materials and Methods

### Ethics Statement

The animal usage protocols were approved by the Institutional Animal Care and Usage Committee at the University of North Dakota (protocol no. 1108-3) and the University of Texas at San Antonio (protocol no. MU066). All the procedures strictly followed the institutional and federal guidelines and all efforts were made to minimize animal suffering.

### Bacterial Strains and Mice

The F.n. strain U112 and an attenuated transposon mutant lacking a 58 kDa protein of hypothetical function (kindly provided by Dr. Larry Gallagher, University of Washington) were grown on Trypticase Soy Agar (TSA) medium supplemented with L-cysteine at 37°C. After overnight growth, the bacteria were harvested and suspended in a freezing medium (250 mM sucrose, 10 mM sodium phosphate pH 7.2 and 5 mM glutamic acid). Stocks were aliquoted and frozen at −80°C for further use.

All *in-vivo* experiments were performed using 6–8 wk old female C57Bl/6 wild-type and galectin-3^−/−^ mice. Galectin-3^−/−^ mice were purchased from Jackson Laboratories (Bar Harbor, ME). Sex- and age-matched galectin-3^+/+^ mice with the same genetic background were used as control.

### Antibodies and Reagents

All reagents were purchased from Sigma-Aldrich unless otherwise indicated. For detection of galectin-3 by immunofluorescence (IF) staining, a purified rat anti-mouse galectin-3 antibody (eBioscience, San Diego, CA) followed by Alexa-546 conjugated chicken anti-rat antibody (Molecular Probes, OR) was used. A rat anti-mouse CD11b antibody conjugated to PE (BD Pharmingen) and a purified rat anti-mouse Gr1 monoclonal antibody, clone Ly-6G (Clone Accurate Chemical, Westbury, NY, USA), followed by the secondary antibody RRX-conjugated Affipure goat anti-rat IgG (Jackson ImmunoResearch Laboratories, West Grove, PA, USA) were used for double staining of activated neutrophils. The terminal deoxyribonucleotidyl transferase-mediated triphosphate (dUTP)-biotin nick end labeling (TUNEL) staining kit was purchased from Chemicon International, CA. Purified recombinant galectin-3 was purchased from R&D Systems, MN. The endotoxin level was <1.0 EU per µg of protein. For detection of reactive oxygen species, Fc OxyBURST assay reagent was purchased from Molecular Probes, Eugene, OR. Mouse IL-6 and TNF-α ELISA kits (BD OptEIA) were from BD Biosciences, San Diego, CA.

### Infection of Mice, Survival and Bacterial Burden

Mice were anaesthetized with a mixture of ketamine HCL and xylazine (30 mg/ml ketamine, 4 mg/ml xylazine in PBS) and were infected intranasally with 50–70 CFUs of the wild-type F.n. strain U112 in 20 µl of PBS or with 20 µl of PBS alone. Mice were monitored daily for signs of disease, which typically included piloerection, hunched gait, lethargy and eye discharge. The survival of infected mice was recorded for up to 2 weeks post-infection (p.i.). Mice displaying severe signs of distress (labored breathing, non-responsiveness to cage tapping, failure of grooming and severe eye discharge) were humanely sacrificed by injecting a mixture of ketamine (90–120 mg/kg) and xylazine (10 mg/kg) followed by cervical dislocation. The death was recorded as tularemia induced mortality. For non-lethal infection, the mice were similarly inoculated with the mutant bacteria followed 3 weeks later by challenge with similar dose of the wild-type organisms. In some experiments, the mice were euthanized at 3 days p.i. and blood, lungs and liver were aseptically harvested. The organs were homogenized aseptically in cold PBS with Complete™ protease inhibitor cocktail (Roche Diagnostics, Germany). For the bacterial burden analyses, the homogenates and blood were serially diluted in PBS and plated on TSA. CFU counts per mouse were calculated after incubating the plates at 37°C overnight.

### Quantitative Real-time PCR

Lungs from infected and mock control mice at various times post-infection were immediately removed after perfusion and total RNA was extracted using Trizol reagent (Invitrogen) according to the manufacturer’s instructions. Real-time PCR analysis of the samples was performed using SYBR green (Applied Biosystems, CA, USA) as the detection dye to measure the expression levels of Galectin-3-specific mRNAs. Briefly, one microgram of total RNA from either infected or mock infected mice was reverse transcribed into cDNA by using a high capacity cDNA reverse transcription kit according to the manufacturer’s instructions (Applied Biosystems, CA, USA). Transcript levels of the housekeeping ribosomal 18S and galectin-3 were PCR amplified in each sample by using specific primers (Advanced Nucleic Acids Core Facility, UTHSCSA, TX): 18S (sense) 5′-CATGTGGTGTTGAGGAAAGCA-3′ and (anti sense) 5′-GTCGTGGGTTCTGCATGATG-3′; Gal-3 (sense) 5′- CAGTGCAGAGGCGTCGGGAAA-3′ and (anti-sense) 5′-CTGCCCCAGCAGGCTGGTTT-3′. The target gene expression levels were normalized to levels of the house keeping 18S gene in the same sample. Expression of galectin-3 in infected samples was determined as fold change over that in control samples as calculated by using the formula 2^−(ΔΔCt)^.

### Histological and Immunofluorescence Staining

For histological and immunofluorescence staining, frozen lung tissues were processed as previously described [3], [9]. Frozen lung sections thus obtained were stained with hematoxylin and eosin for pathological analysis, or for detection of galectin-3 and activated neutrophils (CD11b^+^Gr1^+^) by immunofluorescence staining, as previously described [24]. For detection of cell death, TUNEL method was used according to manufacturer’s instructions (Chemicon International, CA). The images were acquired using a Leica DMR epifluorescent microscope (Leica Microsystems, Wetzlar, Germany) with an attached cooled CCD SPOT RT camera (Diagnostic Instruments Inc., Sterling Heights, MI). The images were processed and analyzed using Adobe Photoshop 7.0 software (Adobe, Mountain view, CA).

### Enumeration of Cellular Infiltration in Lungs

Lungs were harvested from infected and mock control mice at 3 days p.i. after perfusion with PBS and were treated with collagenase to obtain single cell suspensions as previously described [3], [4], [25]. Total numbers of viable immune cells in lungs of infected and mock control galectin-3^−/−^ or WT mice were counted by trypan blue exclusion staining.

### Multi-analyte Profile Analysis

The lung homogenates were prepared as described for the bacterial burden analysis above and were centrifuged at 2000×g for 15 min to clear cellular debris. The supernatants were immediately frozen at −80°C. The biomarker levels in lung homogenates were determined commercially by Myriad Rules-based Medicine (Austin, TX, USA) utilizing a multiplexed flow-based system: Mouse MAP™ (Multi-Analyte Profiles) analysis technology.

### Neutrophil and Macrophage Activation

Cells were isolated from the peritoneal cavities of naïve C57BL/6 mice 12–14 h after intraperitoneal injection with sterile 4% thioglycollate. Neutrophil percentage was determined by flow cytometry using neutrophil specific anti-mouse Gr-1 (anti-Ly-6G and Ly6C). Additionally, the lavage cells were cytocentrifuged on glass slides and were stained with H&E as described above. The cells were plated at the density of 1×10^6^ cells and were infected with wild-type F.n. strain U112 at MOI 50 with or without pretreatment of the cells with 10 µg/ml of purified recombinant galectin-3. Cells stimulated with galectin-3 alone or with 10 ng/ml of phorbol myristate acetate (PMA) served as controls. One hour after stimulation, production of reactive oxygen species (ROS) was measured in the cells by flow cytometry using Fc OxyBURST reagent according to the manufacturer’s instructions. A minimum of 10,000 events was read for each sample and all the cells fluorescing positive in FITC channel (excitation and emission maxima of ∼490 nm and ∼520 nm, respectively) were gated to get the percentage of ROS producing cells.

Bone marrow was isolated from wild-type and galectin-3^−/−^ mice and the cells were differentiated to macrophages as previously described [26]. On day 6 of culture 90–95% cells were macrophages as determined by flow cytometry using macrophage specific markers CD11b and F4/80. The cells were plated at 8×10^4^ cells per well in 96-well flat-bottom plates and were stimulated as described above for the neutrophils. Culture supernatants were collected 24 h after stimulation and measurement of IL-6 and TNF-α was performed by ELISA according to the manufacturer’s instructions (BD OptEIA, BD Biosciences).

### Statistical Analysis

Survival of the infected wild-type and galectin-3^−/−^ mice was compared using Kaplan Meier log Rank test. Statistical comparison between levels of host mediators in different experimental groups was performed by Student’s *t* test using Sigma Plot 8.0.

## Results

### Galectin-3 is Highly Expressed and is Localized Extracellularly in Lungs during the Septic Phase of F.n. Infection

**Figure 1:**
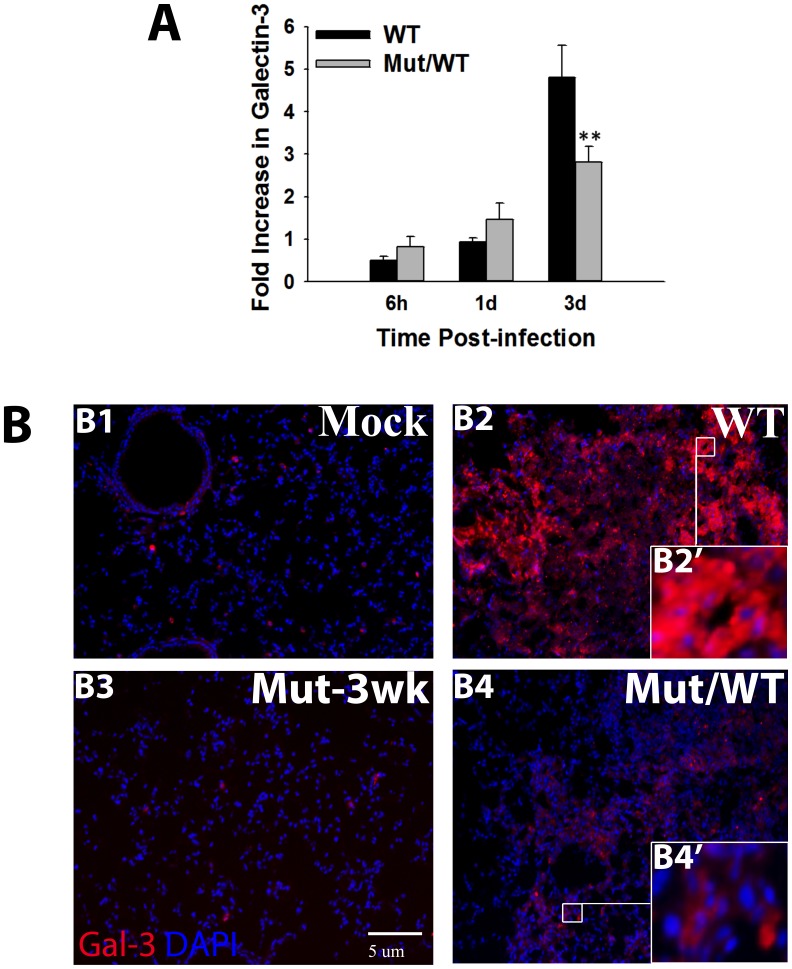
Upregulated expression and extracellular release of Galectin-3 in lungs during respiratory *F. novicida* infection. (**A**) Total RNA was extracted by Trizol method from lungs harvested at the indicated times after infection with the Wild-type bacteria (WT) or from mice vaccinated with an attenuated mutant strain followed by challenge with WT bacteria (Mut/WT mice). The mRNA levels of Galectin-3 were analyzed by real-time PCR as described in Materials and Methods and are expressed as fold changes over the levels in mock control mice. Data shown are the averages of 3–4 mice per group. Statistically significant differences are denoted by asterisks (**p<0.005). (**B**) In-situ IF staining of frozen lung sections from mock infected and WT U112 infected or Mut/WT mice harvested at 3 d. p.i Lung harvested 3 weeks after vaccination with the mutant alone (Mut-3 wk) served as controls for Mut/WT mice. The sections were stained for galectin-3 (red) using a purified rat anti-mouse galectin-3 antibody followed by Alexa-546 conjugated chicken anti-rat antibody. Nuclei (blue) were stained with 4′6′ diamidino-2-phenylindol-dilactate (DAPI). Magnification×200. Insets depict extracellular galectin-3 in WT *F. novicida* infected mouse lungs (B2’) and cytosolic galectin-3 in Mut/WT (B4’) mouse lungs.

Alarmins are characterized as intracellular host factors which display extracellular release under pathological conditions. To examine if galectin-3 exhibits this alarmin property in pulmonary tularemia, the expression and distribution of this lectin was analyzed. We compared the transcript and protein level expression of galectin-3 at various times post-infection (p.i.) in lungs of mice undergoing lethal pulmonary infection with the wild-type strain of F.n. versus the mice vaccinated with an attenuated mutant of F.n. (Mut/WT mice), which protects the mice from sepsis. This mutant has been characterized extensively in our previous studies [3]. As shown in Figure 1A, galectin-3 transcript levels showed maximal increase at 3 days p.i. (dp.i.) in the lungs of mice infected with the wild-type bacteria as well as in the Mut/WT mice. However, this increase was significantly higher in mice undergoing lethal infection as compared to the protected Mut/WT mice. This increase in galectin-3 expression at 3 dp.i. is consistent with the appearance of other sepsis features (extensive cell death, hyperinflammatory response, increased vascular injury) at this time, as shown in our previous studies with F.n. as well as the fully virulent *F. tularensis* [3], [4]. We thus termed this as septic phase of Francisella infection and carried out the rest of our analysis at this time point. Immunofluorescence (IF) analysis of galectin-3 protein expression in frozen sections of lungs harvested at 3dp.i. showed a low basal level expression in mock infected mice inoculated with PBS alone (Fig. 1.B1). Consistent with the transcript data, lungs of mice undergoing septic infection with the wild-type F.n. exhibited upregulated expression of this lectin at 3d p.i. (Fig. 1. B2). This increase in expression was substantially higher than that in Mut/WT mice (Fig. 1.B4). The mice infected with mutant alone for 3 weeks and without challenge with WT bacteria (Mut-3 wk) served as control for the Mut/WT mice. In these mice, galectin-3 was observed to be expressed at low basal level similar to mock control animals (Fig. 1B3). Importantly, most of galectin-3 expressed in septic mice was localized extracellularly in large granuloma-like areas of cellular infiltration, undergoing extensive cell death (Fig. 1.B2’). The non-septic Mut/WT mice, on the other hand, showed intracellular galectin-3 associated with live cells (Fig. 1.B4’). Western blot analysis of bronchoalveolar lavage (BAL) from mice infected with the WT *F. novicida* also showed a significantly high extracellular release of galectin-3 (**Figure S1**). Taken together, these data clearly showed that galectin-3 exhibits a characteristic alarmin property of extracellular release during septic phase of pulmonary F.n. infection.

### F.n. Infected Galectin-3^−/−^ Mice Display Reduced Inflammatory Response and Neutrophil Accumulation

**Figure 2:**
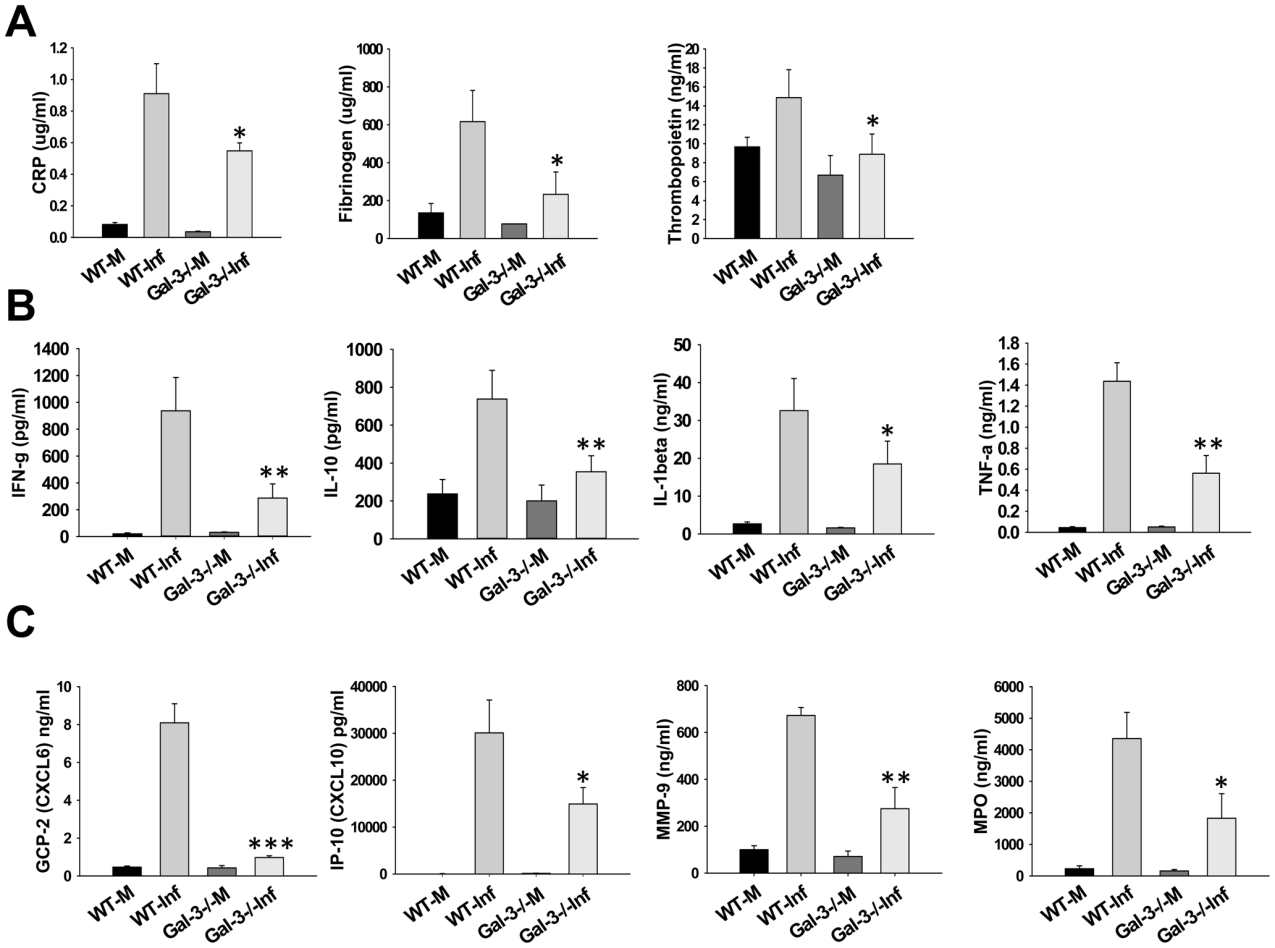
Galectin-3^−/−^ mice display reduced levels of inflammatory mediators in lungs after pulmonary infection with F.n. The lungs from WT mock infected (WT-M), galectin-3^−/−^ mock infected (Gal-3^−/−^M), WT *F. novicida* infected (WT-Inf) or galectin-3^−/−^ *F. novicida* infected mice (Gal3^−/−^Inf) were harvested at 3 d.p.i., homogenized with protease inhibitors in PBS and analyzed commercially for rodent multi-analyte profiles (Rules-Based Medicine, Austin, TX). (**A**), levels of vascular injury markers; (**B**), levels of inflammatory cytokines; and (**C**), levels of neutrophil attractant chemokines and activation markers in lung homogenates. Results shown are from 3–4 mice per group from 3 different experiments. CRP; C-reactive protein, MMP-9; matrix metalloproteinase 9, MPO; myeloperoxidase. *p<0.05; **p<0.005.

**Figure 3:**
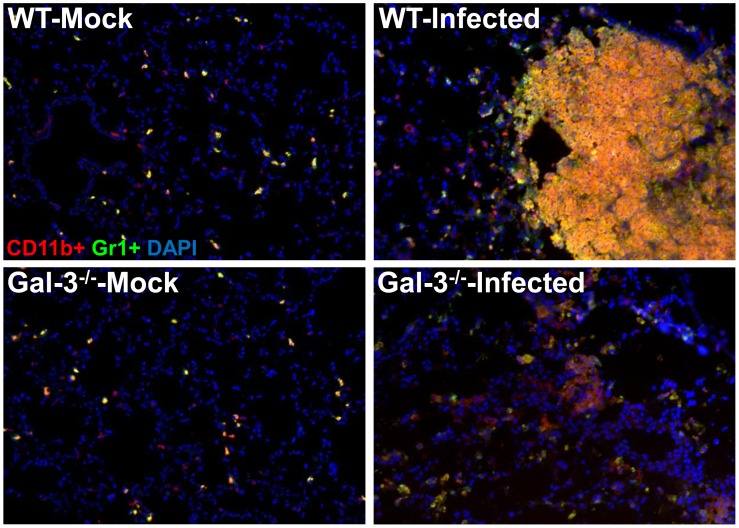
Galectin-3^−/−^ mice display reduced accumulation of neutrophils in lungs during F.n. infection. Frozen sections of lungs harvested at 3 d. p.i. from mock infected and *F. novicida* infected WT or galectin-3^−/−^ mice were co-stained with antibodies against myeloid cell markers CD11b (red) and Gr1 (green). A high co-expression of both markers is depicted by yellow color in infected WT lungs while cells infiltrating lungs of galectin-3^−/−^ mice exhibited expression of only CD11b. Nuclei (blue) were stained with 4′6′ diamidino-2-phenylindol-dilactate (DAPI). Magnification×200. Asterisks depict lesions in the lungs.

We hypothesized that, similar to the function of alarmins, increased expression and extracellular localization of galectin-3 may be contributing to the hyperinflammatory response culminating in sepsis during lethal Francisella infection. In order to analyze this, lungs were harvested at 3d.p.i. from F.n. infected galectin-3^−/−^ and wild-type mice and the levels of multiple cytokines, chemokines as well as vascular injury markers were measured using a multiplex assay. Galectin-3^−/−^ mice displayed significant reduction in levels of several vascular injury markers in comparison with their wild-type counterparts (Fig. 2A). In addition, levels of several inflammatory cytokines (TNF-α, IL-10, IL-1β), described as markers of sepsis, were reduced in galectin-3^−/−^ mice (Fig. 2B). These observations strongly suggested an immune-stimulatory role of galectin-3 during pulmonary Francisella infection. Interestingly, in comparison with wild-type mice, infected galectin-3^−/−^ mice displayed a reduction in several chemokines involved in neutrophil recruitment (Fig. 2C). Furthermore, levels of myeloperoxidase (MPO), a neutrophil associated protease and marker of neutrophil activation, was also reduced in infected galectin-3^−/−^ mice. In order to correlate these observations with cellular infiltration in-vivo, IF staining for co-expression of CD11b and Gr1, markers for activated neutrophils [27], was performed on lung sections from galectin-3^−/−^ and wild-type mice. Consistent with the chemokine data, cells infiltrating the lungs of infected wild-type mice showed high co-expression of CD11b and Gr1, suggesting an activated neutrophil phenotype. These cells were mostly accumulated in large lesion like areas in the lungs of these mice. The cells in infected galectin-3^−/−^ mice, on the other hand, expressed CD11b, but low or no Gr1 (Fig. 3). These results suggested a role of galectin-3 in regulation of myeloid cell accumulation, particularly neutrophils, in the lungs of mice during pulmonary F.n. infection.

### Galectin-3 Regulates F.n. Infection Induced Inflammatory Response In-vitro

**Figure 4:**
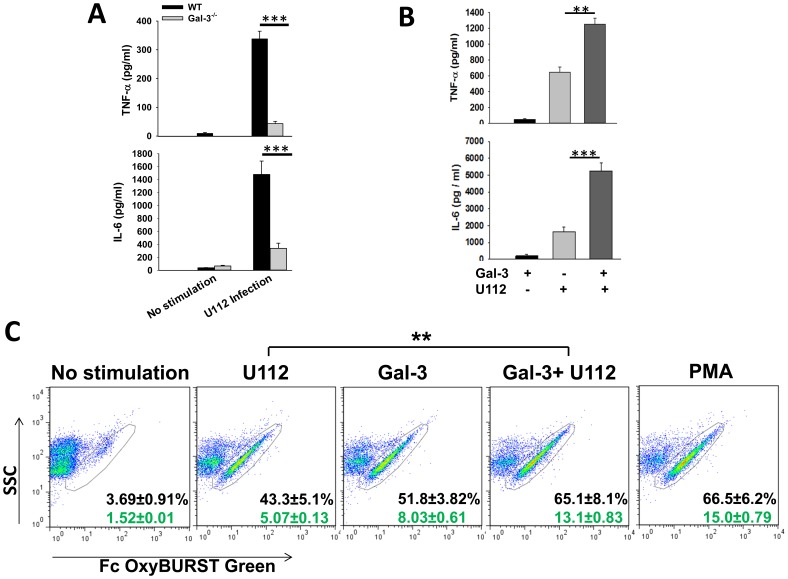
Galectin-3 regulates F.n. infection induced inflammatory response *in-vitro*. (**A**). Bone marrow derived macrophages (BMDMs) were isolated from wild-type and galectin-3^−/−^ mice as described in Methods. The cells were infected with wild-type F.n. Strain U112 at MOI of 50 and the culture supernatents were collected 24 h after infection. The amount of TNF-α and IL-6 were measured in the supernatents by Sandwich ELISA. (**B**). BMDMs from C57Bl/6 wild-type mice were infected with wild-type F.n. Strain U112 at an MOI of 50 with or without pretreatment with 10 µg/ml of purified recombinant galectin-3. Culture supernatants were collected 24 h after infection and the amount of TNF-α and IL-6 were measured by ELISA. The experiment was repeated three times with similar results. (**C**). Peritoneal neutrophils were isolated from mice 12–14 h after injection with 4% thioglycollate and were stimulated with *F. novicida* at an MOI 50 with or without pre-treatment with purified recombinant galectin-3 (10 µg/ml). Stimulation with galectin-3 alone or phorbol myristate acetate (PMA, 10 ng/ml) was used as a control. Production of reactive oxygen species was measured one hour post-stimulation by flow-cytometry using Fc-OxyBURST dye following the manufacturer’s instructions. Numbers in black on the plots depict percent of ROS positive cells and the numbers in green represent median fluorescence intensity (MFI) of individual cells. Dot plots from a representative of 3 independent experiments are shown. Statistical analysis between the data sets was performed by Student’s t test where **p<0.005; ***p<0.001.

In order to further investigate the immune stimulatory properties of galectin-3, we examined the role of this lectin in in-vitro activation of myeloid cells, particularly neutrophils and macrophages. These are the major cell types that infiltrate the lungs of Francisella infected mice [3], [28]. In-vitro infection of WT bone marrow derived macrophages (BMDMs) with wild-type F.n. U112 resulted in an inflammatory response in terms of increased TNF-α and IL-6 production (Fig. 4A). Galectin-3^−/−^ macrophages on the other hand, produced significantly lower amounts of these cytokines in response to infection (Fig. 4A). As the extracellularly released galectin-3 may be playing a role in activation of myeloid cells in-vivo, we examined if pretreatment of these cells with galectin-3 has any effect on Francisella infection induced cytokine production. Stimulation of macrophages with purified galectin-3 induced minimal amount of TNF-α and IL-6 production (Fig. 4B). The optimal concentration of galectin-3 was experimentally determined by using 1–20 µg/ml of the recombinant protein (data not shown). Infection with wild-type F.n. strain U112 infection, on the other hand, induced substantial amounts of these cytokines in macrophages. Interestingly, pre-treatment of macrophages with purified galectin-3 exacerbated this Francisella-induced inflammatory cytokine production (Fig. 4B). Immune stimulatory effect of galectin-3 was also examined on peritoneal neutrophils. Cells collected by peritoneal lavage following intraperitoneal injection of thioglycollate were 80–85% neutrophils as determined by flow cytometry and morphological analysis with characteristic multilobed nuclei (**Figure S2**). Unlike macrophages, treatment of neutrophils with purified galectin-3 alone activated these cells to produce substantial levels of ROS as determined by oxidation of Fc OxyBURST dye (Fig. 4C). Importantly, pre-treatment of neutrophils with this lectin primed these cells to produce further increased amounts of ROS in response to F.n. infection, which was significantly higher than that elicited by F.n. infection alone (Fig. 4C). This cell-type specific response of galectin-3 indicates involvement of distinct receptors and/or signaling pathways, which is currently being investigated in our laboratory. Nonetheless, this augmentation of Francisella infection-induced myeloid cell activation by galectin-3 likely has implications in exacerbation of inflammation culminating in sepsis development during this infection.

### Galectin-3^−/−^ Mice Exhibit Reduced Lung Pathology after F.n. Infection

**Figure 5:**
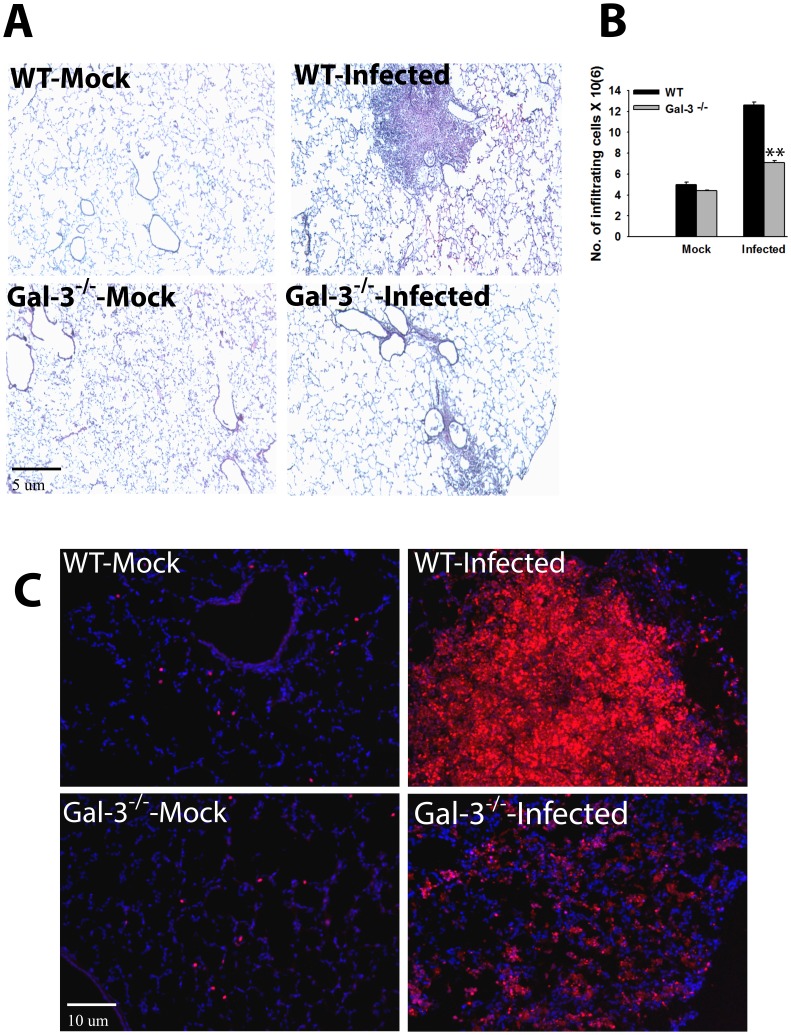
Galectin-3 deficiency leads to improved lung pathology, reduced leukocyte accumulation and reduced cell death upon pulmonary F.n. infection. (**A**) Lungs from mock infected and F.n. infected wild-type (WT) or galectin-3^−/−^ mice were harvested at the septic phase (3 d. p.i.), embedded in optimal-cutting-temperature (OCT) compound, and sectioned as described in Materials and Methods. The frozen sections were stained with Hematoxylin and Eosin. The images obtained are representatives of three experiments performed, and in each experiment each group contained three mice. Magnification, ×200. (**B**). Lungs from mock infected and F.n. infected WT or galectin-3^−/−^ mice were harvested 3 days after intranasal infection. Total immune cells infiltrating the lungs were isolated by collagenase treatment of lungs as described in Materials and Methods. Total numbers of viable immune cells were counted by trypan blue exclusion staining (n = 5–6). Statistical analysis between the data sets was performed by Student’s t test where **p<0.005. (**C**). Frozen lung sections from mock infected and Francisella infected WT or galectin-3^−/−^ mice were processed for in-situ TUNEL staining for detection of DNA fragmentation (red) in nuclei. Nuclei (blue) were stained with 4′,6′-diamidino-2-phenylindole dilactate. Magnification, ×100.

Lung cryosections from wild-type and galectin-3^−/−^ mice infected with a lethal dose of F.n. were stained with H&E and processed for histopathological analyses as described in Materials and Methods. Mock infected wild-type and galectin-3^−/−^ mice exhibited similar normal lung architecture with minimal cellular infiltration and clear air spaces (Fig. 5A). As expected, a massive increase in cellular infiltration and extensive pathology, along with severe bronchopneumonia and massive cell death occurring in the center of large granuloma-like areas of infiltration, was evident in the lungs of wild-type mice at 3 dp.i. (Fig. 5A). The lungs of galectin-3^−/−^ mice, on the other hand, showed moderate peribronchial and perivascular infiltration (Fig. 5A). The infiltrating cells in these areas appeared to be viable and the areas of infiltration lacked cellular debris that is typical of extensive apoptosis and necrosis in the wild-type mice. This was consistent with reduced numbers of leukocytes enumerated after collagenase treatment of the lungs harvested from galectin-3^−/−^ mice (Fig. 5B). Galectin-3 deficiency did not affect the basal number of cells as mock infected wild-type and galectin-3^−/−^ animals showed similar low number of cells in the lungs. To further analyze the extent of cell death in the lungs of infected wild-type and galectin-3^−/−^ mice, TUNEL assay was performed on frozen sections of lungs harvested at 3 dp.i. As shown in Fig. 5C, mock infected wild-type and galectin-3^−/−^ mice showed minimal numbers of TUNEL positive cells in their lungs. On the other hand, septic lungs of F.n. infected wild-type mice showed extensive cell death within perivascular and peribronchial lesions which are the main sites of immune cell infiltration during infection (Fig. 5C). In contrast, the numbers of apoptotic TUNEL positive cells in infected galectin-3^−/−^ mice were much less as compared to their wild-type counterparts following infection with F.n. The improved lung architecture and reduced cell death in the absence of galectin-3 indicates a pathological role of this lectin during pulmonary Francisella infection.

### Galectin-3^−/−^ Mice Show Improved Survival Following F.n. Infection

**Figure 6:**
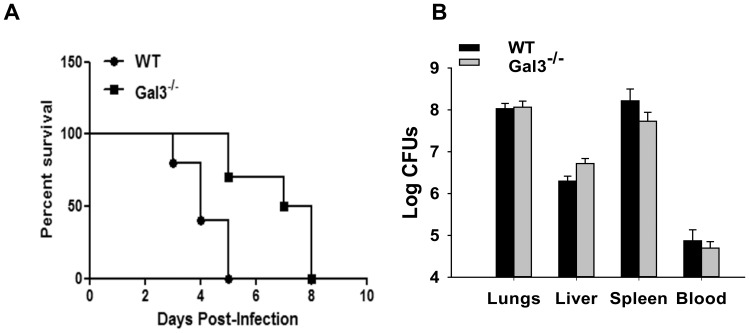
Galectin-3^−/−^ mice show improved survival during pulmonary F.n. infection. (**A**). Fifteen C57Bl/6 WT and 17 galectin-3^−/−^ mice in 3 separate experiments were inoculated intranasally with *F. novicida* and were monitored for survival daily for 2 weeks. The improved survival of galectin-3^−/−^ mice compared to WT mice was statistically significant, as determined by Kaplan-Meier log-rank analysis (*P* value*** = 0.0003). (**B**) Bacterial burdens in lungs harvested from *F. novicida* infected WT and galectin-3^−/−^ mice at 3 d.p.i. Lungs and liver were harvested from infected mice, homogenized as described in Materials and Methods. Tissue homogenates and blood harvested at the same time, were serially diluted and plated on TSA plates to enumerate bacterial burdens. In this representative of three independent experiments, each group contained three to five mice.

In order to see the effect of improved lung pathology and reduced inflammatory responses in the absence of galectin-3, overall disease severity and survival was compared in C57BL/6 wild-type and galectin-3^−/−^ mice infected with a lethal dose of F.n. In the infected wild-type mice, visible signs of disease started to appear by day 3 p.i. which typically included piloerection, hunched gait, lethargy, and eye discharge. All of these mice succumbed to infection by day 5 p.i. (Fig. 6A). By contrast galectin-3^−/−^ mice exhibited delayed appearance of disease symptoms and showed significantly improved survival as compared to the infected wild-type mice (Fig. 6A). Intriguingly, enumeration of bacterial burden in the organs of these mice at the peak of infection, i.e., 3 d.p.i. showed that both galectin-3^−/−^ and the wild-type animals exhibited similar bacterial burdens in their systemic organs as well as in blood (Fig. 6B).

## Discussion

Sepsis is the 2^nd^ leading cause of death in ICU patients and pulmonary infections in turn are a major source of sepsis [29]. It is a complex immune disorder resulting from deregulation of multiple host defense pathways. Accumulating evidence suggests that host endogenous molecules termed alarmins, likely play an important role in pathophysisology of sepsis [30]. In this study we show that galectin-3, a mammalian β-galactoside binding lectin acts as a novel alarmin in development of sepsis during pulmonary infection with *F. novicida*. Consistent with characteristic properties of alarmins, galectin-3 was upregulated and extracellularly released during the septic phase of infection and could amplify the Francisella infection-induced inflammatory response of neutrophils and macrophages. Furthermore, galectin-3^−/−^ mice showed improved pathology, reduced inflammation and improved survival during pulmonary Francisella infection. These results suggest that galectin-3 functions as an alarmin and plays a pathogenic role in development of sepsis in pulmonary bacterial infection.

Alarmins are structurally diverse multifunctional host proteins with some common properties (12–15). These are endogenous proteins performing homeostatic functions that lack any signal sequence for active secretion and have chemoattractant and immune activating properties, once released in extracellular milieu. With advances in our understanding of host responses to pathogenic events, the list of alarmins has continued to grow over the past decade. Several well characterized alarmins such as High Mobility Group Box1 (HMGB-1), S100 family of proteins, and heat shock proteins have been shown to perform dual functions as factors controlling homeostatic processes like transcriptional regulation when localized to intracellular compartments and as pro-inflammatory factors upon their release from necrotic cells during a pathogenic insult [31]–[33]. Similarly, galectin-3 when localized in the nucleus, has been shown to act as an RNA splicing factor [34] and performs homeostatic functions such as embryogenesis and cell cycle regulation [19]. The results of current study showed that galectin-3 can be released extracellularly in lungs under pathogenic conditions such as an infection. Curiously, galectin-3 does not contain any signal sequence for golgi mediated classical secretion. Thus active secretion of this lectin is likely via a yet unclear non-classical secretion pathway, a property shared by most alarmins characterized to date [20]. However, in F.n. infection, this lectin is likely released passively from dead/dying cells since the extracellular galectin-3 is detected only during lethal infection with F.n., which typically results in extensive cell death [3], [4], [7]. On the other hand, the mice vaccinated with an attenuated F.n. mutant causing little or no cell death showed this lectin to be intracellular and largely associated with live cells, with minimal levels in BAL. Notwithstanding the mechanism involved, release of galectin-3 in the septic phase of F.n. infection indicates a role for this molecule in pathogenesis of this infection.

Consistent with alarmin properties, galectin-3 exhibited immune activating properties such as stimulation of oxidative burst in neutrophils and inflammatory cytokine production in macrophages. Importantly, this lectin was able to augment F.n. infection induced inflammatory response from neutrophils as well as macrophages, which can have important implications under in-vivo conditions. Previous studies from our laboratory have shown that pulmonary infection with F.n., as well as *F. tularensis*, results in extensive cell death and that F.n. infected phagocytes are defective in efferocytosis, the process of clearing dead cell debris [11]. Coinciding with this, we have further shown that pulmonary Francisella infection is characterized by hyperinflammatory response with an unbridled increase in levels of several inflammatory cytokines as well as vascular injury markers [3], [7]. It is likely that during pulmonary Francisella infection, galectin-3 released from these dead cells primes the bystander myeloid cells to produce heightened levels of inflammatory mediators in response to the bacteria, resulting in further tissue damage and ultimately organ failure, characteristic of sepsis. Curiously, while the macrophages did not respond to galectin-3 alone, neutrophils displayed ROS production upon stimulation with galectin-3 or F.n. infection alone, which was amplified upon combination of the two. This cell-specific nature of galectin-3 activity in the context of Francisella infection is interesting and is in line with a previous study showing the role of prototype alarmin HMGB1 in promoting the inflammatory response of monocytes elicited by external stimuli [35]. Intriguingly, in that study as well, the monocytes did not respond to HMGB1 alone. In light of several biologically distinct functions performed by alarmins ranging from inflammation to tissue repair and wound healing, association with other stimuli possibly adds another layer to the regulation of their mechanisms of action. Although speculative at this stage, it is possible that the activating receptor for galectin-3 on neutrophils is constitutively expressed while that on macrophages likely gets expressed in an infection-specific manner. Further studies to test this hypothesis are currently underway in our laboratory.

As mentioned earlier, one of the characteristic properties of alarmins is to mediate immune cell influx. The reduced number of leukocytes in infected galectin-3^−/−^ animals in this study indicates that this lectin likely plays a role in recruitment of these cells in sepsis. Reduced levels of neutrophil chemoattractants and activation markers in galectin-3^−/−^ mice support this notion. This observation is in line with the role of prototypic alarmin HMGB1 in neutrophil migration by regulating the levels of chemoattractants such as IL-8 [36]. Additionally galectin-3 may also play a direct role in extravasation of neutrophils from blood vessels into the lungs, as shown in a previous study with pulmonary *S. pneumonia* infection [37]. Other proinflammatory alarmins such as S100 proteins have also been shown to directly promote migration of myeloid cells by binding to their cell surface receptors [38], [39]. Another possibility is that galectin-3 may have a role in inhibiting neutrophil turnover. Under the conditions of a resolving inflammatory response to an infectious insult, once the neutrophils have performed their antimicrobial function, they undergo programmed cell death. The failure of this process is the root cause of several inflammatory disorders, as prolonged exposure to neutrophilic factors can result in non-specific tissue damage [40]. In this regard a recent study showed a defect in neutrophil turnover and thus the lack of resolution of inflammation during Francisella infection [41]. Furthermore, a previous study has shown the involvement of galectin-3 in decreasing neutrophil death as well as reducing macrophage cell death in response to apoptotic stimuli [42], [43]. This suggests that expression of galectin-3 in inflammatory cells may lead to their enhanced survival, resulting in exacerbated inflammation. Thus it is tempting to speculate that galectin-3, in addition to activating neutrophils, may be playing a role in inhibition of neutrophil turnover during Francisella infection by prolonging the lifespan of these cells. Consistent with this hypothesis, F.n. infected galectin-3^−/−^ mice show reduced numbers of neutrophils, lower levels of neutrophil associated immune mediators and consequently reduced tissue pathology. These observations further support proinflammatory and pathogenic role of galectin-3 in pulmonary F.n. induced sepsis.

Extensive tissue pathology is a major complication of acute respiratory infections which are associated with severe sepsis [44], [45]. This is caused by hyper activation of the inflammatory immune response that results in capillary leakage, tissue injury, and ultimately lethal organ failure [46], [47]. Results from the current study showed that F.n. infected galectin-3^−/−^ animals exhibited a reduction in the levels of sepsis mediators such as vascular injury markers thrombopoietin, fibrinogen, as well as acute phase protein CRP and inflammatory cytokines such as TNF-α, IL-6 and IL-1. This observation further suggested that galectin-3 mediates upregulation of these sepsis markers during Francisella infection and thus plays a role in development of sepsis. As a result of this mitigated inflammatory response and tissue pathology, galectin-3^−/−^ mice are able to survive the infection for a significantly longer duration as compared to infected wild-type mice. Galectin-3^−/−^ mice have been shown to be highly resistant to infection with another Gram negative bacterium Salmonella [21] with KO mice exhibiting lower bacterial burdens. Although the mechanism of this protection was not shown, it was speculated that galectin-3 binds to and masks bacterial PAMPs resulting in immune suppression and unchecked bacterial growth. Previous studies have shown the ability of galectin-3 to bind to a variety of pathogens [48]–[51] owing to its specificity for β-galactosides which are a common constituent on pathogen membranes. This interaction can serve to activate immune response as well as result in direct killing of the pathogen as shown in case of Candida [50]. Since we observed similar bacterial burdens in *F. novicida* infected galectin-3^−/−^ and wild-type mice, it seems unlikely that galectin-3 is involved in direct killing of bacteria. It is, however, possible that galectin-3 could be binding to a Francisella factor thus potentiating its interaction with immune activating receptor/s, as has been shown in case of HMGB1 [35]. Studies regarding the identity of Francisella factors possibly engaged by galectin-3 and immunological consequences of these interactions are currently on-going in our laboratory. The survival advantage of *F. novicida* infected galectin-3^−/−^ was observed to be only transient as these mice ultimately succumbed to infection, possibly due to overwhelming bacterial burdens. Interestingly, this survival advantage of galectin-3^−/−^ mice was dependent on the infection dose of bacteria. The galectin-3^−/−^ mice succumbed to the infection at a similar rate as WT mice when infected with 300–500 CFUs of bacteria (data not shown). It is possible that at that dose, a higher bacterial burden leads to a further increase in cell death leading to an increased accumulation of other alarmins which mask the advantageous effect of the absence of galectin-3. This observation highlights the complex nature of sepsis syndrome where multiple host and pathogen derived factors cross talk and regulate various immune pathways. It is also consistent with previous studies showing partial or no protection upon blocking single alarmin such as HMGB1 [52]–[54]. Nonetheless, as the bacteria can be cleared by successful antibiotic therapy, the complications often arise from tissue damage during sepsis. Thus, a combinatorial approach using blockage of galectin-3 along with antibiotics could prove to be a successful therapy for treating Francisella infection induced sepsis.

In Toto, our findings indicate that galectin-3 plays a pathogenic role as an alarmin to exacerbate the inflammatory response during pulmonary infection with Francisella and contributes to sepsis development. Galectin-3 thus may represent a potential target for treatment of sepsis during this infection.

## Supporting Information

Figure S1 **Upregulated expression and extracellular release of Galectin-3 in lungs during respiratory F. novicida infection.** Bronchoalveolar lavage (BAL) was obtained from lungs of mice infected with the wild-type F. novicida strain U112 or PBS alone as previously described (9). Galectin-3 was immunoprecipitated from BAL using a purified rat anti-mouse galectin-3 antibody (eBioscience, San Diego, CA) by previously described method [55] with modifications. Briefly 1 mg of total BAL proteins were incubated with 10 µg anti-galectin-3 antibody at 4°C overnight. Immune complexes were pulled down with using 30 µl of 30% Protein A Plus agarose beads (Pierce) for 2 h at 4°C. The beads were washed, solubilized in 1× SDS gel loading buffer and resolved on 12% acrylamide gels (BioRad). The gels were processed for western blotting as described previously [3] for detection of galectin-3 using anti-mouse galectin-3 antibody. Densitometric analysis of bands was performed using the Lumi-Imager software (Roche Applied Science). Bar graph depicts densitometry analysis of galectin-3 bands represented in arbitrary units. Statistically significant differences are denoted by asterisks (***p<0.001).
(TIF)

Figure S2 **Flow cytometry analysis of peritoneal neutrophils.** Mice were injected intraperitoneally with sterile 4% thioglycollate. Peritoneum was lavaged 12–14 hrs later and cells were analyzed by flow cytometry using neutrophil specific anti-mouse Gr1 (Ly6G+Ly6C) antibody. In addition, cells were cytocentrifuged and stained with H&E for morphological analysis.
(TIF)
