# Supplementary figures and images for: Galectin-3 Functions as an Alarmin: Pathogenic Role for Sepsis Development in Murine Respiratory Tularemia
Source: PLoS One. 2013 Mar 20;8(3):e59616. doi: 10.1371/journal.pone.0059616 (PMC3603908; doi:10.1371/journal.pone.0059616)

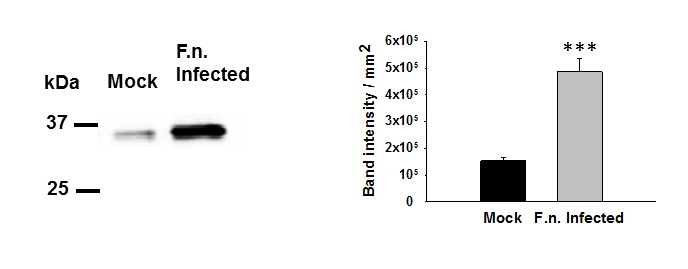

Supplement: Figure S1 — Upregulated expression and extracellular release of Galectin-3 in lungs during respiratory F. novicida infection. Bronchoalveolar lavage (BAL) was obtained from lungs of mice infected with the wild-type F. novicida strain U112 or PBS alone as previously described (9). Galectin-3 was immunoprecipitated from BAL using a purified rat anti-mouse galectin-3 antibody (eBioscience, San Diego, CA) by previously described method [55] with modifications. Briefly 1 mg of total BAL proteins were incubated with 10 µg anti-galectin-3 antibody at 4°C overnight. Immune complexes were pulled down with using 30 µl of 30% Protein A Plus agarose beads (Pierce) for 2 h at 4°C. The beads were washed, solubilized in 1× SDS gel loading buffer and resolved on 12% acrylamide gels (BioRad). The gels were processed for western blotting as described previously [3] for detection of galectin-3 using anti-mouse galectin-3 antibody. Densitometric analysis of bands was performed using the Lumi-Imager software (Roche Applied Science). Bar graph depicts densitometry analysis of galectin-3 bands represented in arbitrary units. Statistically significant differences are denoted by asterisks (***p<0.001). (TIF) [file pone.0059616.s001.tif]

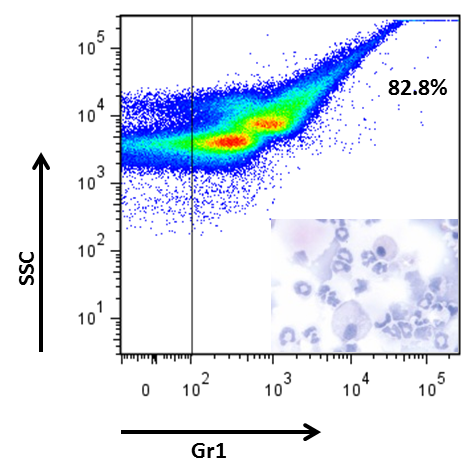

Supplement: Figure S2 — Flow cytometry analysis of peritoneal neutrophils. Mice were injected intraperitoneally with sterile 4% thioglycollate. Peritoneum was lavaged 12–14 hrs later and cells were analyzed by flow cytometry using neutrophil specific anti-mouse Gr1 (Ly6G+Ly6C) antibody. In addition, cells were cytocentrifuged and stained with H&E for morphological analysis. (TIF) [file pone.0059616.s002.tif]
